# Expression of CD226 is upregulated on Tr1 cells from neuromyelitis optica spectrum disorder patients

**DOI:** 10.1002/brb3.2623

**Published:** 2022-05-19

**Authors:** Ping Chen, Mingmei Wu, Ning Wang, Feng Xia, Fang Du, Zhirong Liu, Jinchun Wang, Jingyi Jin, Boquan Jin, Gang Zhao, Lihua Chen, Jing Yi, Liang Fang

**Affiliations:** ^1^ Department of Neurology Xianyang Hospital of Yan'an University Xianyang China; ^2^ Department of Immunology Medical College of Yan'an University Yan'an China; ^3^ Department of Immunology the Fourth Military Medical University Xi'an China; ^4^ Department of Neurobiology the Fourth Military Medical University Xi'an China; ^5^ Department of Neurology Xijing Hospital the Fourth Military Medical University Xi'an China; ^6^ Department of Transfusion Medicine Xijing Hospital the Fourth Military Medical University Xi'an China

**Keywords:** biomarker, CD226, NMOSD, Tr1 cell

## Abstract

**Background:**

Neuromyelitis optica spectrum disorder (NMOSD) is a central and acute demyelinating disease of the central nervous system with unusual clinical course. The development of novel biomarkers for NMOSD is critical for implementing effective clinical treatment. CD226 is known to be expressed on many types of peripheral lymphoid cells. However, the expression level and function of CD226 on type 1 T regulatory (Tr1) cells during NMOSD is unknown.

**Methods:**

Eighteen patients with NMOSD and 10 healthy volunteers were enrolled in the test group to probe the difference of CD226 expression on Tr1 cells using flow cytometric analysis.

**Results:**

The expression of CD226 on Tr1 cells exhibited significantly increased tendency in NMOSD patients. Additionally, methylprednisolone and rituximab treatment decreased the expression of CD226 on Tr1 cells. Furthermore, the expression of CD226 on Tr1 cells was correlated with disease severity.

**Conclusion:**

This study provides a new basic insight into CD226 expression pattern on Tr1 cells, which have great potential to be biomarkers for monitoring the development and treatment of NMOSD.

## Graphical abstract

CD226 on Tr1 cells was higher in NMOSD patients. The expression of CD226 on Tr1 cells was correlated with disease severity. CD226 on Tr1 cells might be biomarkers of NMOSD.

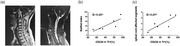

## 1 INTRODUCTION

Neuromyelitis optica spectrum disorder (NMOSD) includes the entity previously known as neuromyelitis optica (NMO) and patients with limited forms (e.g., only myelitis or optic neuritis) and comprises a phenotypic continuum of primarily immune‐mediated astrocyte injury, rather than a primary demyelinating disease, with preferential involvement of the optic nerves, brainstem, and the spinal cord (de Seze et al., 2016; Franciotta et al., 2017). A considerable advancement in the understanding of those disorders was the identification of pathogenic autoantibodies against aquaporin‐4 (anti‐AQP4‐IgG) in patients with NMO, which allowed for the establishment of NMO as a distinct nosological entity (Fujihara & Misu, 2015; Jurynczyk et al., 2017; Prain et al., 2019). Despite the fact that the majority of patients with NMOSD are seropositive for autoantibodies against AQP4, there are adaptive T‐cell autoimmune responses in NMOSD, but further in‐depth mechanism is unclear.

Regulatory T cells are subsets of cells involved in the immune response and autoimmune responses in vivo. They have immunomodulatory or immunosuppressive functions and have an important role in maintaining peripheral immune tolerance. The number and function of such T cells are associated with the development of autoimmune diseases. Regulatory T cells are mainly Foxp3^+^ Treg cells and Foxp3^–^ Tr1 cells (Sakaguchi et al., 2010; Zeng et al., 2015). Foxp3^+^ Treg cells are characterized by constitutive expression of CD4, CD25, and Foxp3. Coexpression of CD49b and LAG‐3 (CD223) selectively identifies both human and murine Tr1 cells. Both of the cells can dampen autoimmunity and tissue inflammation partly through their secretion of the immunosuppressive cytokine IL‐10 (Neumann et al., 2019). CD226, an important costimulatory molecule for T cells, is widely involved in the regulation of immune cells, such as the differentiation and effector function of initial T cells and the polarization and effector functions of Th1 cells (Dardalhon et al., 2005; Sherrington et al., 1997). However, there are few studies on how CD226 affects the occurrence and development of human NMOSD. It is reported that the expression levels of inducible costimulatory molecule (ICOS) and programmed death‐1 (PD‐1) in the peripheral CD4^+^ T cells of patients with NMOSD were significantly higher than those of the normal control group, showing that costimulatory molecules may play important roles in NMOSD pathogenesis (Xue et al., 2019).

Our laboratory has previously shown that CD226 polyclonal antibody treatment of EAE mice has reduced symptoms and susceptibility by promoted IL‐10 production (Zhang et al., 2016). Therefore, we speculate that CD226 may affect the occurrence and development of EAE by participating in the regulation of regulatory T cells. In this study, we aimed to identify the specific expression of CD226 in regulatory T cells of NMOSD patients. These may provide useful information to help us understand the pathogenesis and outcome of NMOSD.

## 2 MATERIALS AND METHODS

### 2.1 Study population

**FIGURE 1:**
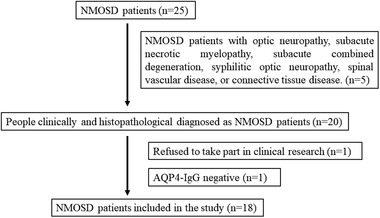
Flow diagram of patient selection

Eighteen patients with NMOSD and ten healthy volunteers were retrospectively analyzed from January 1, 2015 to December 31, 2018 in Xijing Hospital. Our study was approved by the Human Ethics Committee of Xijing Hospital, Fourth Military Medical University. Informed consent was obtained from each participant. The diagnosis criteria are as follows: (1) AQP4‐IgG positive; (2) at least one core symptom, such as optic neuritis, long‐segment acute myelitis, extreme posterior syndrome, acute brain stem syndrome, and acute encephalic clinical syndrome. People with optic neuropathy, subacute necrotic myelopathy, subacute combined degeneration, syphilitic optic neuropathy, spinal vascular disease, and connective tissue disease were not included in the study (Figure 1). The details of the clinical presentation, signs and symptoms, and relevant laboratory findings are summarized in Table S1.

### 2.2 Medication

Six patients were treated with 1 g of methylprednisolone (halved every 3 days, ending in 27 days) and low‐dose Rituxi mAb at 100 mg and immunosuppressant, such as azathioprine or mycophenolate (50 mg, multiplied every 3 days, until 200 mg, bid.po) and/or not IVIG 0.4g/kg.

### 2.3 Flow cytometric analysis

Peripheral blood mononuclear cells (PBMCs) were isolated from EDTA anticoagulation peripheral blood by Ficoll density gradient centrifugation and washed twice with PBS. Tr1 cells were stained for surface markers using anti‐CD3 APC‐cy7, anti‐CD4 Percp‐cy5.5, anti‐CD226 APC, anti‐CD49b FITC, anti‐CD223 PE for 30 min. Treg cells were intracellular stained by anti‐CD3 APC‐cy7, anti‐CD4 Percp‐cy5.5, anti‐CD226 APC, anti‐CD25 PE, and anti‐Foxp3 FITC according to the manufacturer's instruction (all from BD Biosciences, San Jose, CA, USA). Samples were washed twice with staining buffer, and then analyzed by flow cytometry using a FACSAria flow cytometer.

### 2.4 Statistical analysis

Data representing the means ± standard deviation (SD) were compared by nonparametric Mann–Whitney U tests to determine significance. Pearson correlation coefficients were calculated for CD226^+^Tr1 cells to Barthel index score and spinal cord affected segments. All analyses were performed using GraphPad Prism version 6.0 (GraphPad Software, San Diego, CA).

## 3 RESULTS

### 3.1 Tr1 and Treg cells decreased in NMOSD patients

**FIGURE 2:**
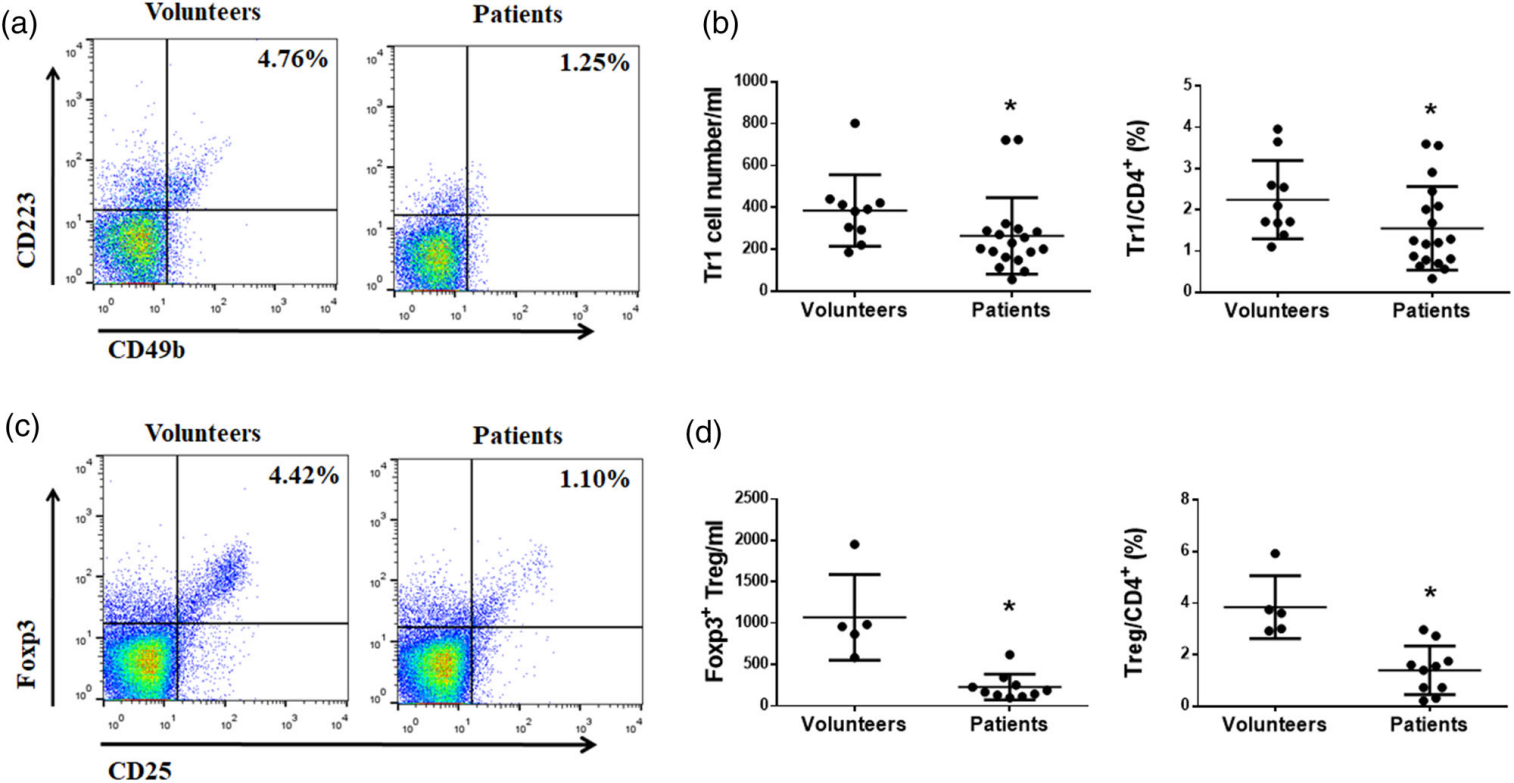
Tr1 and Treg cells decreased in peripheral blood of patients with NMOSD. (a) Peripheral blood mononuclear cells (PBMCs) from NMOSD patients and healthy volunteers were analyzed by staining with CD3, CD4, CD223, and CD49b. Numbers indicate the percentage of Tr1 cells. (b) The percentage and absolute number of Tr1 cells were significantly decreased in NMOSD patients. (c) Peripheral blood mononuclear cells (PBMCs) from NMOSD patients and healthy volunteers were analyzed by staining with CD3, CD4, Foxp3, and CD25. Numbers indicate the percentage of Foxp3^+^ Tregs. (d) The percentage and absolute number of Foxp3^+^ Tregs were significantly decreased in NMOSD patients (*p < .05)

It is reported that Treg cells are involved in the development of autoimmune diseases, and there are few reports on the changes in the number of Tr1 and Foxp3^+^Treg cells in NMOSD diseases. In this study, we first examined the changes of Tr1 cells in the peripheral blood of NMOSD patients. Eighteen patients and ten healthy volunteers were enrolled. NMOSD patients had lower number of Tr1 cells (CD4^+^CD49b^+^CD223^+^) (Gagliani et al., 2013), and Tr1 cells/CD4^+^ T cells ratio was also reduced (Figure 2a,b). We further compared Foxp3^+^Treg cell numbers in 10 patients and 5 healthy volunteers. Foxp3^+^Treg cells in the peripheral blood of NMOSD patients were markedly reduced, and Foxp3^+^Treg cells/CD4^+^ T cells ratio was also lower compared with healthy volunteers (Figure 2c,d). These results indicate that both Tr1 and Foxp3^+^Treg cells might be involved in the pathogenesis of NMOSD.

### 3.2 The expression of CD226 on Tr1 cells was higher in NMOSD patients

**FIGURE 3:**
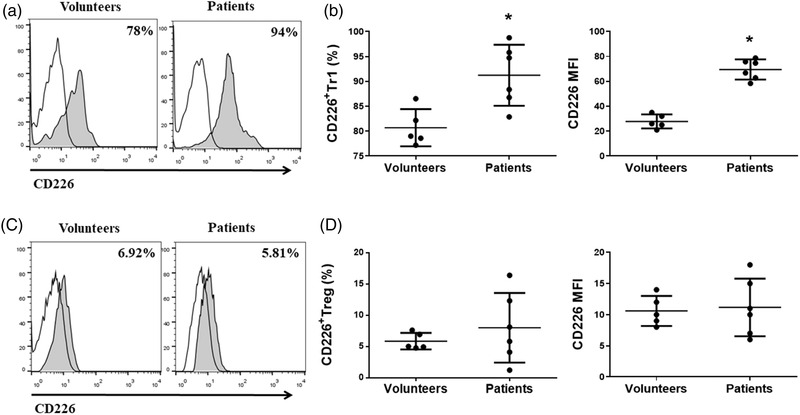
The expression of CD226 on Tr1 cells was increased in NMOSD patients. (a) Expression level of CD226 in gated Tr1 cells in peripheral blood were evaluated by FACS analysis. Numbers indicate the percentage of CD226^+^ Tr1 cells. (b) The percentage of CD226^+^Tr1 cells was significantly increased in NMOSD patients. CD226 mean fluorescence intensity (MFI) was quantified on the right. (c) Expression level of CD226 in gated Treg cells in peripheral blood were evaluated by FACS analysis. Numbers indicate the percentage of CD226^+^ Treg cells. (d) The percentage of CD226^+^Treg cells in NMOSD patients and healthy volunteers. CD226 MFI was quantified on the right (*p < 0.05)

It was shown that human Tr1 cells express high level of CD226, but its function has not been confirmed in Tr1 cells. In this study, we assessed the expression of CD226 on Tr1 cells from six NMOSD patients and five healthy volunteers. The percentage and mean fluorescence intensity (MFI) of CD226 on Tr1 cells of NMOSD patients was significantly higher than that in healthy control group (Figure 3a,b). In contrast, we did not observe a statistically significant difference in the levels of CD226 on Foxp3^+^Treg cells of NMOSD patients compared with healthy volunteers (Figure 3c,d).

### 3.3 Tr1 and Treg cells increased in NMOSD patients after treatment

**FIGURE 4:**
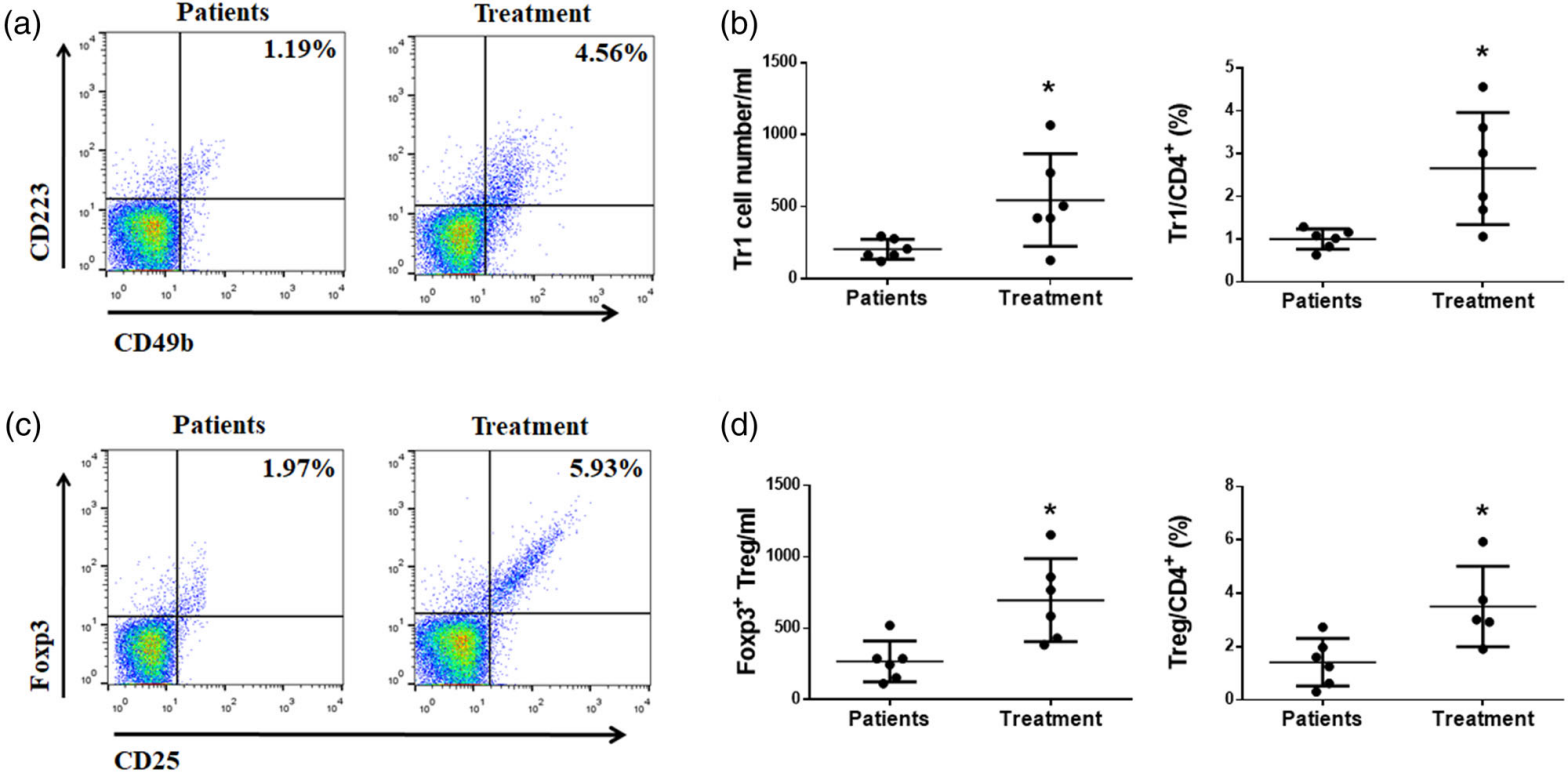
Foxp3^+^Tregs and Tr1 cells were significantly recovered after the treatment. (a) Peripheral blood mononuclear cells (PBMCs) from NMOSD patients and healthy volunteers were analyzed by staining with CD3, CD4, CD223, and CD49b. Numbers indicate the percentage of Tr1 cells. (b) The percentage and absolute number of Tr1 cells were significantly increased in NMOSD patients after the treatment. (c) Peripheral blood mononuclear cells (PBMCs) from NMOSD patients and healthy volunteers were analyzed by staining with CD3, CD4, Foxp3, and CD25. Numbers indicate the percentages of Foxp3^+^ Tregs. (d) The percentage and absolute number of Foxp3^+^ Tregs were significantly increased in NMOSD patients after the treatment (*p < 0.05)

Next, we examined Tr1 and Foxp3^+^Treg cell numbers in six NMOSD patients before and after treatment. NMOSD patients were treated with 1 g of methylprednisolone and 100 mg of rituximab for 1 month. We found that NMOSD patients showed increased cell number of Tr1 cells after treatment, and Tr1 cells/CD4^+^ T cells ratio was also elevated (Figure 4a,b). Moreover, NMOSD patients with treatment showed a higher number of Foxp3^+^Treg cells and Foxp3^+^Treg cells/CD4^+^ T cells ratio in the peripheral blood (Figure 4c,d).

### 3.4 The expression of CD226 on Tr1 cells decreased in NMOSD patients after treatment

**FIGURE 5:**
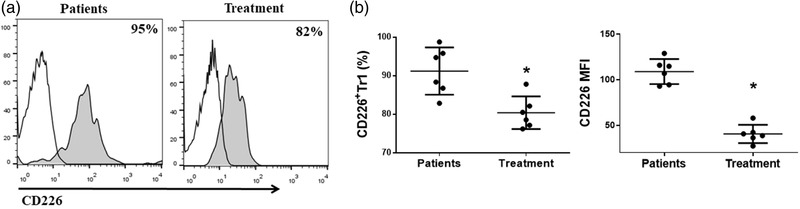
The expression of CD226 on Tr1 cells was decreased in NMOSD patients after the treatment. (a) Expression level of CD226 in gated Tr1 cells in peripheral blood was evaluated by FACS analysis. Numbers indicate the percentage of CD226^+^ Tr1 cells. (b) The percentage of CD226^+^Tr1 cells was significantly decreased in NMOSD patients after the treatment. CD226 MFI was quantified on the right (*p < 0.05)

Assuming that CD226 was involved in the pathogenesis of NMOSD, we compared the expression of CD226 on Tr1 and Foxp3^+^Treg cells after methylprednisolone and rituximab treatment. We found the percentage and MFI of CD226 on Tr1 cells was significantly decreased after treatment (Figure 5a,b), while there was no significant difference in the expression of CD226 on Treg cells (data not shown). These data indicated that CD226 might be important for Tr1 cell function in NMOSD.

### 3.5 The expression of CD226 on Tr1 cells was correlated with disease severity

**FIGURE 6:**
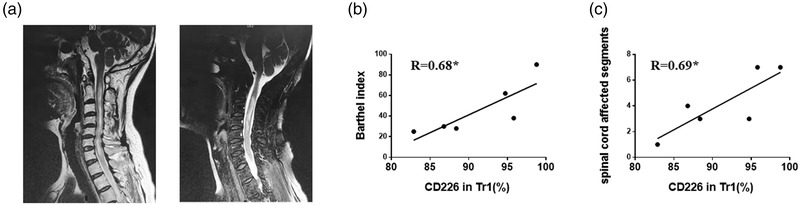
Correlation analyses between the expression of CD226 on Tr1 cells and disease severity. (a) MRI show abnormal signals in the spinal cord in NMOSD patients. (b) The correlation analyses between the expression of CD226 on Tr1 cells and Barthel index scores. (c) The correlation analyses between the expression of CD226 on Tr1 cells and spinal cord affected segments (*p < 0.05)

We analyzed the association between CD226 expression level on Tr1 cells from NMOSD patients and Barthel index scores or spinal cord affected segment. MRI showed abnormal signals in the spinal cord of NMOSD patients (Figure 6a). We found that the frequencies of CD226 on Tr1 cells were positively correlated with Barthel index scores (Figure 6b). Interestingly, they were also positively correlated with disease severity (Figure 6c). Enhanced spinal cord affected segment was observed in NMOSD patients with higher expression of CD226 on Tr1 cells. These results indicate that the proportion of CD226 on Tr1 cells of NMOSD patients may be associated with disease progression.

## 4 DISCUSSION

CD226 is an immunoglobulin‐like molecule expressed in hematopoietic cells such as T cells, NK cells, a subset of B cells, monocytes/macrophages, and platelets in human (Castriconi et al., 2004; Li et al., 2020; Nagayama‐Hasegawa et al., 2019; Sherrington et al., 1997; Vo et al., 2016). Previous studies examining the role of CD226 have primarily focused on the function of intercellular adhesion, lymphocyte signaling, and cytotoxicity induced by T lymphocytes and NK cells. It has recently become appreciated that CD226 might be involved in functions of regulatory T cells (Fourcade et al., 2018; Fuhrman et al., 2015; Mu et al., 2020; Wang et al., 2019). Our previous study showed CD226 pAb administration in vivo reduced the onset of EAE in mice by promoting IL‐10 production and regulating T cell differentiation (Zhang et al., 2016). Moreover, the CD226^+^TIGIT^–^ T cell population was associated with reduced Treg purity and suppressive capacity after expansion (Fuhrman et al., 2015), and CD226 deficiency on Treg cells can aggravate renal fibrosis (Mu et al., 2020).

NMOSD is an uncommon antibody‐mediated disease of the central nervous system. NMOSD was stratified by AQP4‐antibody serostatus (Franciotta et al., 2017; Fujihara & Misu, 2015; Prain et al., 2019). There was no controversy over AQP4‐antibody‐seropositive NMOSD, but alternative diagnoses were required with AQP4‐antibody‐seronegative (Fujihara, 2019; Jurynczyk et al., 2017). There are adaptive T‐cell autoimmune responses in NMOSD. Tr1 cells and Foxp3^+^ regulatory T cells are highly immune‐suppressive subsets of CD4^+^ T cells, and they exert important suppressive and homeostasis effects on cancer, autoimmune disorders, and inflammatory responses (Jia et al., 2019; Josefowicz et al., 2012; Kleinewietfeld & Hafler, 2014; Roncarolo et al., 2014, 2018; Zeng et al., 2015). Considering previous findings reporting the number of Tr1 cells was decreased, and the activity of Tr1 cells for secreting IL‐10 was also impaired in MS patients, MS monkeys, and mouse EAE model by multiple mechanisms (Astier & Hafler, 2007; Ma et al., 2009; Martinez‐Forero et al., 2008), we found both Tr1 and Treg cells decreased in NMOSD patients. The abnormal regulatory T cells are also reported in other kinds of autoimmune diseases (Jia et al., 2019; Kleinewietfeld & Hafler, 2014). We previously demonstrated that CD226 impaired Treg suppressive function via CTLA‐4 and TIGIT during EAE (Wang et al., 2019). We analyzed the expression of CD226 on Foxp3^+^Tregs and Tr1 cells in NMOSD patients. Tr1 cells expressed higher levels of CD226 as compared to Tregs in both healthy donors and patients. More importantly, the expression of CD226 on Tr1 cells exhibited significantly increased tendency in NMOSD patients. However, there was no notable difference in the levels of CD226 expression in Tregs. These findings showed CD226 on Tr1 cells may be a good indicator of NMOSD.

Additionally, all NMOSD patients should be treated with immunosuppressive drugs to prevent further attacks. In this study, NMOSD patients were treated with methylprednisolone and rituximab. The treatment reduced the general symptoms, accompanied by the increased Tr1 and Treg cells. In particular, a pronounced decrease of CD226 on Tr1 cells was found, showing that CD226 may be a good indicator of effectiveness of pharmacotherapies. A more comprehensive correlation analysis between CD226 expression and disease severity showed CD226 on Tr1 cells were positively correlated with Barthel index scores. Meanwhile, they were also positively correlated with spinal cord affected segments. While this study has shown new insights into CD226 on Tr1 and Treg cells in NMOSD, the causes that initiate the alteration of CD226 need further investigation.

In summary, our current study detected CD226 expression on Tr1 and Treg cells in NMOSD patients for the first time. Besides, we also found the correlations between CD226 expression on Tr1 cells and NMOSD severity. This study provides pivotal information to understand the mechanisms implicated in NMOSD and inspires new ideas for diagnosis and treatment of the diseases by considering the individual conditions based primarily on the CD226 expression analysis.

## CONFLICT OF INTEREST

The authors declare no conflict of interest.

### PEER REVIEW

The peer review history for this article is available at https://publons.com/publon/10.1002/brb3.2623

## Supporting information

Table S1 General phenotype of patients with NMOSD

## Data Availability

The data that support the findings of this study are available from the corresponding author upon reasonable request.
