# Supplementary material for: Expression of CD226 is upregulated on Tr1 cells from neuromyelitis optica spectrum disorder patients
Source: Brain Behav. 2022 May 19;12(6):e2623. doi: 10.1002/brb3.2623 (PMC9226801; doi:10.1002/brb3.2623)
Supplement: Supplementary file 1 — Table S1 General phenotype of patients with NMOSD [file BRB3-12-e2623-s001.docx]

**Table S1 General phenotype of patients with NMOSD**

| Gender (m:f) |  | | 5:13 | |
| --- | --- | --- | --- | --- |
| Age studied (median, range) | | |  | 41.0 (17-66) |
| **Presenting symptom** | | |  |  |
| Vision loss | | |  | 5 |
| Limb numbness and weakness | | |  | 12 |
| Urinary dysfunction | | |  | 6 |
| **CSF** (ﬁrst performed) | | |  |  |
| Lumber pancture pressure (median, range mmH_2_O) | | |  | 157 (100-245) |
| Pleocytosis (median, range cells/mm^3^) | | |  | 7 [22, (0-196)] |
| Elevated protein (median, range g/L) | |  | | 5 [0.64, (0.16-2.1)] |
| Normal | | |  | 7 |
| **Evoked potential** | | |  |  |
| Visual evoked potential | | |  | 12 |
| Brainstem auditory evoked potential | | |  | 3 |
| Somatosensory evoked potential | | |  | 3 |
| **MRI** (ﬁrst performed) | | |  |  |
| Brain T_2_ /Flair hyperintensities  Frontal lobe, parietal lobe, temporal lobe  Thalamus  corpus callosum  periventricular  Semi-oval center | | |  | 2  2  2  5  3 |
| basal ganglion | | |  | 2 |
| brainstem | | |  | 6 |
| Cervical spinal | | |  | 13 |
| Thoracic spinal | | |  | 8 |
| Lumbar spinal | | |  | 1 |
